# Brassinosteroid-mediated regulation of agronomic traits in rice

**DOI:** 10.1007/s00299-014-1578-7

**Published:** 2014-03-26

**Authors:** Cui Zhang, Ming-yi Bai, Kang Chong

**Affiliations:** 1 Institute of Genetics and Development Biology, Chinese Academy of Sciences, Beijing, 100101 China; 2 The Key Laboratory of Plant Cell Engineering and Germplasm Innovation, Ministry of Education, School of Life Sciences, Shandong University, Jinan, 250100 China; 3 Institute of Botany, Chinese Academy of Sciences, Beijing, 100093 China

**Keywords:** Brassinosteroids, Signaling, Homeostasis, Rice agronomic traits, Grain yield

## Abstract

*****Key message***:**

**Brassinosteroids have important roles in plant development. This review focuses on the agronomic traits regulated by brassinosteroids in rice.**

**Abstract:**

Brassinosteroids (BRs) are a group of steroid phytohormones with wide-ranging biological activity. Genetic, genomic and proteomic studies have greatly advanced our understanding of BR signaling in *Arabidopsis* and revealed a connected signal transduction pathway from the cell surface receptor kinase BRASSINOSTEROID-INSENSITIVE1 (BRI1) and BRI1-ASSOCIATED RECEPTOR KINASE 1 (BAK1) to the BRASSINAZOLE-RESISTANT1 (BZR1) family of transcription factors and their targets mediating physiological functions. However, compared with the dicot model plant *Arabidopsis*, much less is known about BR signaling in rice, which is a monocot. In this review, we provide an update on the progress made by BR studies in rice and discuss how BR regulates various important agronomic traits to determine rice grain yield. Specifically, we discuss the function of novel components including LEAF AND TILLER ANGLE INCREASED CONTROLLER (LIC), DWARF and LOW-TILLERING (DLT), DWARF1 (D1) and TAIHU DWARF1 (TUD1) in rice BR signaling, and provide a rice BR-signaling pathway model that involves a BRI1-dependent pathway as well as a G-protein α subunit-mediated signaling pathway. The recent significant advances in our understanding of BR-mediated molecular mechanisms underlying agronomic traits will be of great help for rice molecular breeding.

## Introduction

Rice (*Oryza sativa* L.) is one of the most important food crops and feeds more than half of the world population. Increasing crop yield is a major challenge for modern agriculture. Rice plant architecture is crucial for grain yield and is determined by plant height, leaf angle, tiller number, and panicle morphology. Phytohormones such as brassinosteroids (BRs) play important roles in development of plant architecture, and rice BR-related mutants such as *d61*, *d2*, *BR*-*deficient dwarf 1* (*brd1*), and *dlt* show dwarfism and erect leaves (Yamamuro et al. 2000; Hong et al. 2005; Tong et al. 2009). This suggests that manipulation of BR biosynthesis or signaling to modify rice architecture could be a feasible approach for improving rice yield.

Brassinosteroids are a class of plant-specific steroidal hormones that are structurally related to animal and insect steroids. Brassinolide (BL), the most biologically active and the naturally occurring form of the BRs was first isolated and purified from *Brassica napus* pollen in Grove et al. (1979). Since then, more than 50 BL analog have been identified (Fujioka and Sakurai 1997). BRs have been shown to regulate various biological processes in plants including seed germination, stomata formation, vascular differentiation, plant architecture, flowering, male fertility and senescence in *Arabidopsis* (Clouse and Sasse 1998). Exogenously applied bioactive BRs increase the resistance of plants to a variety of stresses, including both biotic and abiotic stresses (Li 2010). BRs have widely been used since the 1980s to increase yield in crops and vegetables. In rice, BRs affect many agricultural traits that influence grain yield, including plant height, leaf angle, grain size, and tiller number (Ikekawa and Zhao 1991; Khripach et al. 2000).

Extensive genetic, genomic and proteomic studies have identified all major BR-signaling components in *Arabidopsis* and elucidated the cross talk of BR with other hormonal and environmental signals. Numerous reviews have described current knowledge about the molecular structure of the BR-signaling network (Clouse 2011; Tong and Chu 2012; Vriet et al. 2013; Wang et al. 2012). Compared with the tremendous progress of BR-signaling studies in *Arabidopsis*, much less is known in the monocot model plant rice (Hao et al. 2013). Recently, several important BR-signaling components have been identified in rice, and functional studies of these genes provide promising tools for molecular breeding with modification of the BR-signaling network. In this review, we will briefly introduce BR biosynthesis and signaling in *Arabidopsis* and then focus on BR functional studies in rice.

### BR signaling in *Arabidopsis*

**Fig. 1:**
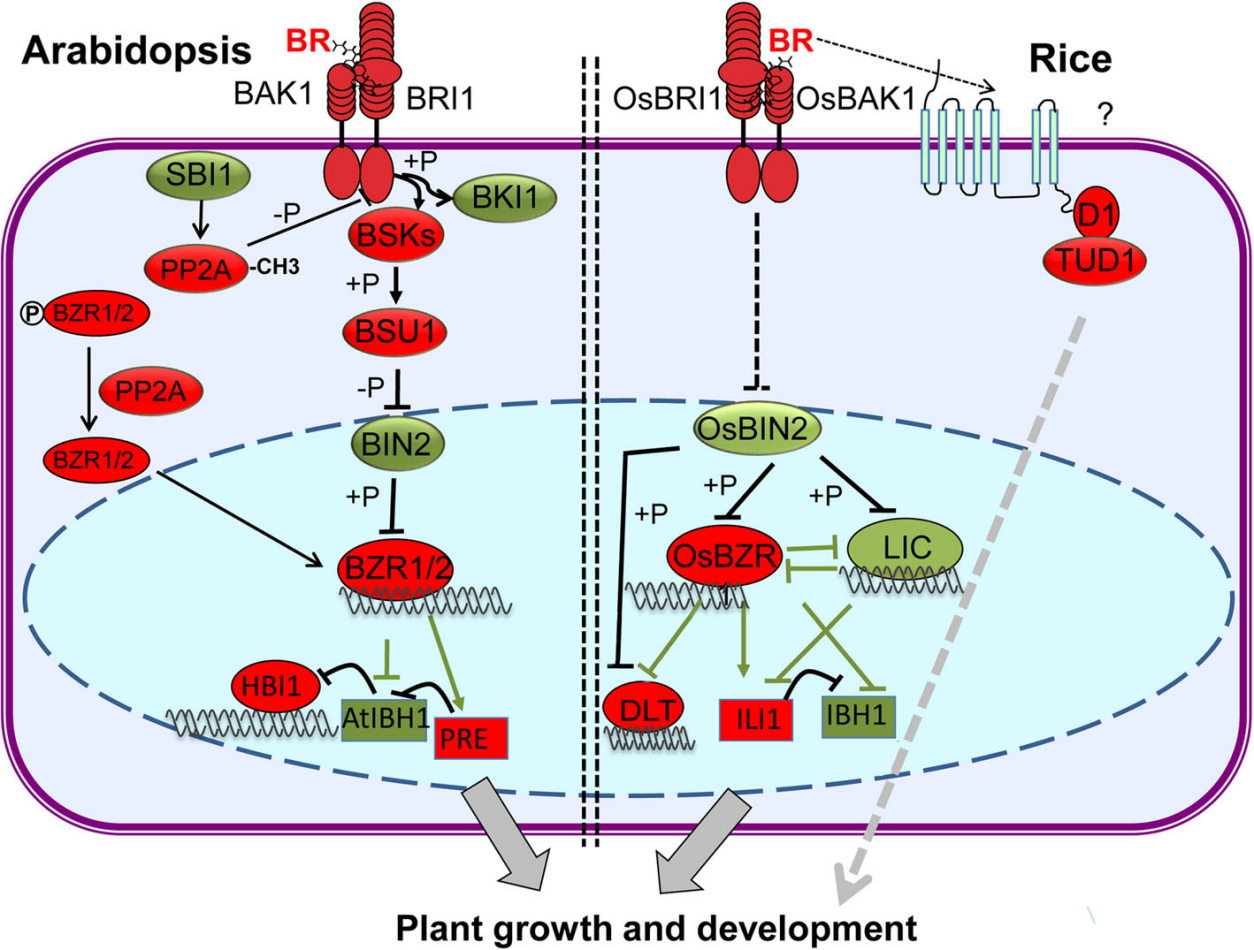
BR-signaling pathways in rice and Arabidopsis. The *left panel* shows the BR signal transduction pathway in *Arabidopsis*. BR directly interacts with BRI1 and BAK1 to form the BRI1–BR–BAK1 complex. Sequential transphosphorylation between BRI1 and BAK1 activates BRI1, which then phosphorylates BSKs/CDG1. The active BSKs/CDG1 phosphorylates BSU1, which dephosphorylates and inactivates BIN2. BZR1 and BES1/BZR2 are dephosphorylated by PP2A and move into the nucleus to induce expression of *PRE1*, but repress *IBH1* expression. PRE1, IBH1 and HBI1 form an antagonistic cascade to regulate plant growth. SBI1 (SUPPRESSOR OF BRI1) is involved in the deactivation of BRI1 through methylation of PP2A. The *right panel* shows the BR-signaling pathway in rice. BR binding to OsBRI1 promotes association with OsBAK1, and inactivates OsBIN2 via an unknown mechanism. OsBIN2 phosphorylates OsBZR1, LIC and DLT, and inhibits their activity. OsBZR1 induces the expression of *ILI1*, but represses the expression of *IBH1*, *LIC* and *DLT*; whereas LIC represses the expression of OsBZR1. The heterotrimeric G-protein α subunit known as D1/RGA1 in rice is involved in another BR-signaling pathway in which D1/RGA1 and TUD1 act together to mediate BR signaling. *Red* indicates the components play positive roles in BR signaling, whereas *green* denotes the components that play negative roles

The signal transduction pathway for BRs from perception to downstream regulation has been extensively studied (Wang et al. 2012). In the absence of BRs, BRI1 KINASE INHIBITOR 1 (BKI1) prevents the heterodimerization of BRI1 with BRI1-ASSOCIATED RECEPTOR KINASE 1 (BAK1) to inactivate BRI1 (Fig. 1) (Jaillais et al. 2011; Wang and Chory 2006). In the presence of BRs, BRs directly interact with the LRR domains of BRI1 and SOMATIC EMBRYO-GENESIS RECEPTOR KINASE (SERK) to form a BRI1–BR–SERK complex, and induce BRI1 and SERK transphosphorylation of each other to activate the BRI1 signaling pathway (Hothorn et al. 2011; Kinoshita et al. 2005; Li and Chory 1997; Li et al. 2002; Nam and Li 2002; Santiago et al. 2013; She et al. 2011; Wang et al. 2008a, b). Activated BRI1 phosphorylates BKI1 on Tyr211, leading to the disassociation of BKI1 from the plasma membrane. Phosphorylated BKI1 interacts with 14-3-3 proteins and releases its inhibition of BZR1 and BZR2 (also named BES1 for BRI1-EMS-SUPPRESSOR 1) (Wang et al. 2011). Activated BRI1 phosphorylates BR-SIGNALING KINASEs (BSKs) and CONSTITUTIVE DIFFERENTIAL GROWTH 1 (CDG1), which subsequently activate the BRI1-SUPPRESSOR 1 (BSU1) phosphatase (Kim et al. 2009, 2011; Mora-Garcia et al. 2004; Tang et al. 2008). BSU1 inactivates BRASSINOSTEROID-INSENSITIVE 2 (BIN2) through dephosphorylation, thus relieving BIN2’s suppression of BZR1 and BZR2/BES1 (He et al. 2002; Li and Nam 2002; Wang et al. 2002; Yin et al. 2002). Upon BR-induced inactivation of BIN2, BZR1 and BES1 are rapidly dephosphorylated by PHOSPHATASE 2A (PP2A) (Tang et al. 2011). Dephosphorylated BZR1 and BES1 dissociate from 14-3-3 proteins and accumulate in the nucleus to regulate the expression of more than one thousand downstream genes (He et al. 2005; Sun et al. 2010; Yin et al. 2005). Yeast two-hybrid and pull-down assays demonstrated that BZR1 interacts with a cyclophilin, CYP20-2, which alters the conformation and enhances phosphorylation of BZR1 (Zhang et al. 2013). BZR1 and BES1 can both regulate BR-related genes directly or through interaction with other transcription factors, such as BES1-INTERACTING Myc-LIKE1 (BIM1) and INTERACT-WITH-SPT6 (IWS) (Li et al. 2010; Yin et al. 2005). Recent studies have revealed that BR-induced SUPPRESSOR of BRI1 (SBI1) methylates PP2A, and activated PP2A dephosphorylates BRI1 to attenuate BR signaling (Fig. 1) (Di Rubbo et al. 2011; Wu et al. 2011).

Although the framework of the BR-signaling network has been established and genome-wide analysis has provided a global view of BR function, most of the BR response targets and their links with other hormones and environmental signals still remain to be characterized (Hao et al. 2013). In *Arabidopsis*, the BR-signaling pathway interacts with signaling of other hormones such as GAs (Bai et al. 2012), ABAs (Zhang et al. 2009b; Zhang et al. 2011), and Strigolectone (Wang et al. 2013) to regulate plant growth and development as well as responses to stress (Table 1).

**Table 1:** The full names of the genes that appear in this review

Abbreviation	Full name
BRI1	BRASSINOSTEROID-INSENSITIVE1
SERK	SOMATIC EMBRYO-GENESIS RECEPTOR KINASE
BAK1	BRI1-ASSOCIATED RECEPTOR KINASE 1
BKI1	BRI1 KINASE INHIBITOR 1
BSK	BR-SIGNALING KINASE
CDG1	CONSTITUTIVE DIFFERENTIAL GROWTH 1
BSU1	BRI1-SUPPRESSOR 1
BIN2	BRASSINOSTEROID-INSENSITIVE 2
BZR1	BRASSINAZOLE-RESISTANT1
BES1	BRI1-EMS-SUPPRESSOR 1
PP2A	PHOSPHATASE 2A
SBI1	SUPPRESSOR of BRI1
IWS1	INTERACT-WITH-SPT6
BIM1	BES1-INTERACTING Myc-LIKE1
LIC	TILLER ANGLE INCREASED CONTROLLER
DLT	DWARF and LOW-TILLERING
TUD1	TAIHU DWARF1
ILI1	Increase Leaf Inclination 1
IBH1	ILI1 Binding bHLH 1
PRE1	Paclobutrazol-resistant 1
HBI1	Homolog of BEE2 Interacting with IBH1
D1	DWARF1
D2	DWARF2
D11	DWARF11
BRD1	BR-deficient dwarf 1
BRD2	BR-deficient dwarf 2
DWF1	DWARF1
DWF4	DWARF4
CPD	Constitutive Photomorphogenesis and Dwarf
DET2	De-Etiolated-2
ROT3	CYP90C1

### BR signaling in rice

Whereas BR biosynthesis and signaling are well understood in *Arabidopsis*, in rice a few components have been characterized either by forward or reverse genetics (Fig. 1). For some BR components such as OsBRI1, OsBAK1, OsGSK1 and OsBZR1, rice orthologs of the known *Arabidopsis* genes have been found, and play conserved functions in the two species. However, orthologous components of *Arabidopsis* BR signaling such as PP2A, BSKs and BSU1 have not been identified in rice up to now. Other components including OsLIC, OsDLT and OsTUD1 have been identified in rice but have no orthologs in *Arabidopsis*, indicating that there are some BR functions specific to rice.

The first identified rice BR-insensitive mutant, *d61*, was defective in an orthologous gene of BRI1. Loss-of-function mutants of OsBRI1, *d61*-*1* and *d61*-*2* show BR insensitivity, erect leaves, dwarf culms, abnormal skotomorphogenesis, and disorganized microtubule arrangement in cells from the non-elongated internodes, suggesting BRs play an important role in rice development (Li et al. 2009; Nakamura et al. 2006; Wang et al. 2007; Yamamuro et al. 2000).

The rice genome contains four genes homologous to *BAK1*, among which *OsBAK1* is the closest relative of *BAK1*. OsBAK1 can directly interact with OsBRI1, and overexpression of *OsBAK1* in transgenic plants causes BR hypersensitivity and partly suppresses both *Arabidopsis* *bri1*-*5* and rice *d61*-*1* mutant phenotypes, suggesting that BAK1 has conserved functions in rice and *Arabidopsis* (Wang et al. 2007; Li et al. 2009).

OsGSK1 is a BIN2 homolog and is involved in BR signaling and stress responses (Koh et al. 2007). Overexpression of *OsGSK1* in *Arabidopsis* leads to plant dwarfism, similar to the phenotype of the gain-of-function mutant *bin2*-*1*. The T-DNA knockout mutant of *OsGSK1* shows greater tolerance of cold, heat, salt and drought stresses, indicating that OsGSK1 has important functions in stress responses.

OsBZR1 is the closest ortholog of BZR1 and BES1, and functions as a positive regulator of BR signaling. Suppression of *OsBZR1* by RNAi leads to erect leaves, semi-dwarfism and BR-insensitive phenotypes. Similarly to in *Arabidopsis*, 14-3-3 proteins bind phosphorylated OsBZR1 to retain OsBZR1 in the cytoplasm (Bai et al. 2007). BZR1 and BES1 directly regulate more than one thousand genes to control multiple biological processes (Sun et al. 2010; Yu et al. 2011). This highlights the need for genome-wide analysis of OsBZR1-regulated genes to elucidate how BR could act through OsBZR1 to regulate yield-determining traits.

OsILI1 and OsIBH1 interact with each other to regulate rice leaf angle antagonistically. OsBZR1 directly binds to the promoter of these two genes to induce *OsILI1*, but represses *OsIBH1* expression (Zhang et al. 2009a, b). *AtPRE1* and *AtIBH1* were identified through searching for orthologous genes of *OsILI1* and *OsIBH1*, respectively, in *Arabidopsis*. Analogously to their orthologs in rice, AtPRE1 binds and inhibits AtIBH1 activity to regulate cell elongation. ILI1/PRE1 and IBH1 belong to the helix–loop–helix (HLH) family, members of which lack the basic domain required for DNA binding but dimerize with DNA-binding basic HLH (bHLH) transcription factors. Homolog of BEE2 Interacting with IBH1 (HBI1) is a positive regulator of cell elongation downstream of the BR, GA, light and temperature signaling pathways. HBI1 binds to DNA containing G-box element, and IBH1 binding inhibits HBI1 DNA-binding activity, whereas PRE1 interacts with IBH1 to prevent its inhibition of HBI1 (Bai et al. 2012). The conserved function of ILI1/PRE1 and IBH1 in rice and *Arabidopsis* suggests that it could be fruitful to test whether the rice ortholog of HBI1 is involved in the BR-signaling pathway to control leaf bending.

**Fig. 2:**
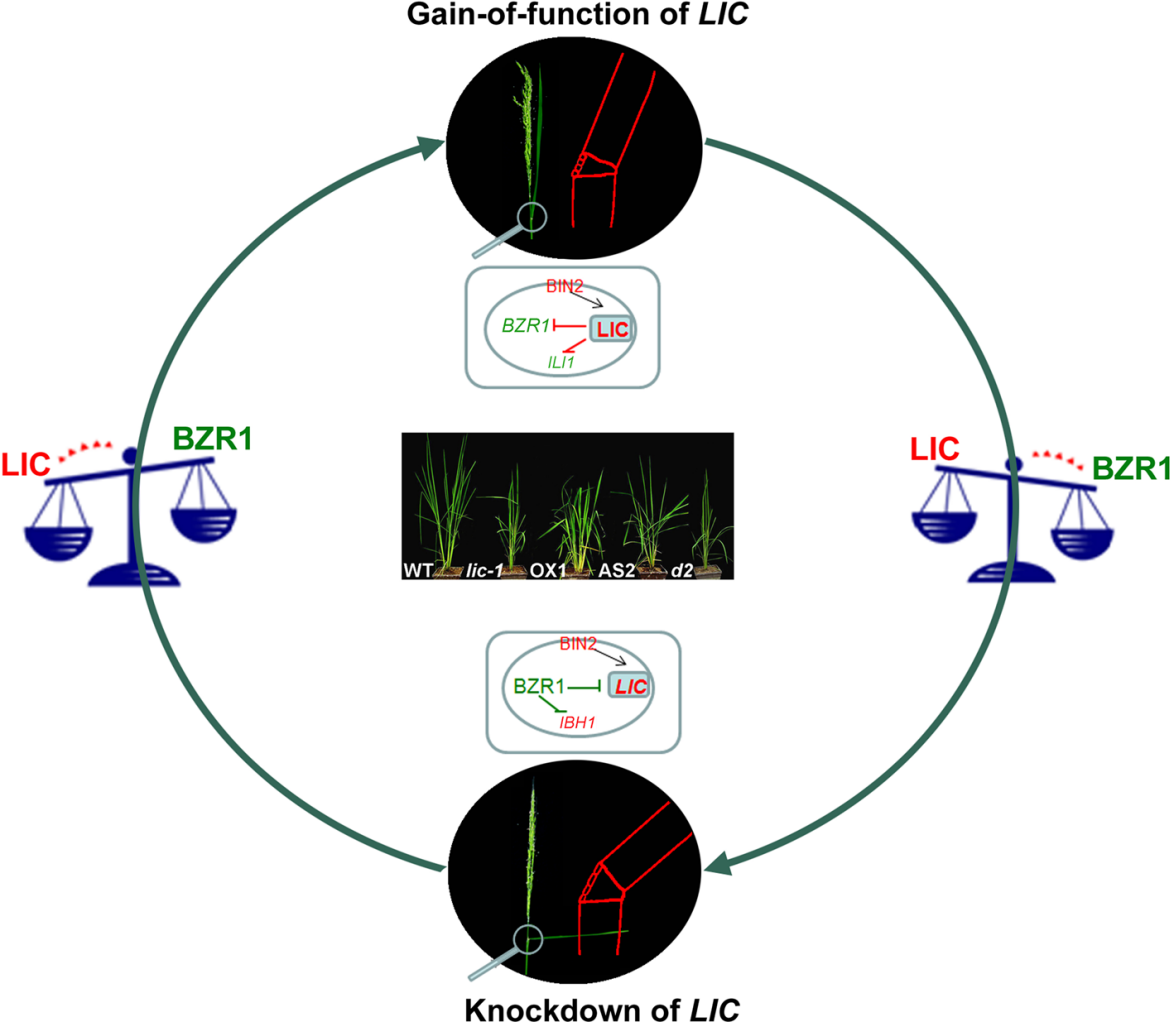
A diagram of LIC antagonism of BZR1 to balance rice BR signaling. The images in the *black circles* show the leaf bending of the gain-of function *LIC* mutant and the antisense line for *LIC.* The *red sketches* show the pulvinus (*triangle-like*, linker between the sheath and leaf) mediating leaf bending. Interior to those images are schematic diagrams of cells, including the nucleus, showing *LIC* regulation. The *LIC* overexpression line 1 (OX1) and the gain-of-function mutant (*lic*-*1*) display erect leaves similar to the BR-biosynthesis mutant *d2*; the *LIC* antisense line 2 (AS2) shows inclined leaves. LIC is a negative regulator of BR signaling while BZR1 is a positive regulator. LIC represses its targets, such as *BZR1* and *ILI1*, on a transcriptional level and inhibits adaxial cell elongation of the pulvinus in the *lic*-*1* mutant and the overexpression lines. This causes the plants to show erect leaves. In *LIC* antisense lines, BZR1 function is dominant. BZR1 represses its targets, such as *LIC* and *IBH1*, to promote cell elongation. The plants therefore show increased leaf angle. LIC and BZR1 antagonize each other to balance BR dynamics during development

OsLIC, encoding a novel CCCH-type zinc finger protein, was identified on the basis of its predominant expression in the stem node. Antisense-mediated suppression of *OsLIC* leads to large leaf and tillering angles, semi-dwarfism, and decreased numbers of seeds in each panicle, whereas a gain-of-function mutant of *OsLIC* displays erect leaves and reduced BR sensitivity. Like OsBZR1, OsLIC interacts with OsGSK1 and is phosphorylated by OsGSK1 for retention in the cytoplasm. BR treatment inhibits OsGSK1 activity, thereby inducing OsLIC dephosphorylation and transfer of OsLIC to the nucleus. Nuclear-located OsLIC directly binds to the *OsBZR1* promoter, which contains CTCGC motifs, and represses *OsBZR1* transcriptional activity. *OsLIC* is a direct target of OsBZR1; knockdown of *OsBZR1* leads to upregulation of *OsLIC*. Thus, OsLIC and OsBZR1 represent a pair of antagonistic transcription factors that repress each other during transcription, and their repression strength may depend on the BR level. *OsBZR1* and *OsLIC* have different expression patterns: OsBZR1 acts in the presence of low levels of BR to promote signaling, whereas OsLIC is predominantly activated by high levels of BR to brake BR signaling. The dynamics of BR responses in rice development are thus modulated by positive regulator OsBZR1 and negative regulator OsLIC (Fig. 2) (Wang et al. 2008a; Zhang et al. 2012a).

The rice *dlt* mutant was identified through screening T-DNA insertion dwarf mutants with similar phenotypes to known BR-related mutants. The *dlt* mutant displays a semi-dwarf and compact stature, dark-green erect leaves, late flowering, and reduced tillering number (Tong et al. 2009). The *dlt* mutant is insensitive to BR in lamina joint bending assays, whereas overexpression of *OsDLT* leads to hypersensitivity to BR. Genetic analysis showed that *dlt* enhances the dwarf phenotype of the BR-deficient mutant *d11*-*2* and the BR-insensitive mutant *d61*-*1*, and overexpression of *OsDLT* rescues the lamina-inclination phenotype of *d61*-*1* and *OsGSK2* antisense lines, suggesting OsDLT acts downstream of OsBRI1 and OsGSK2 to positively regulate BR responses. OsDLT physically interacts with and is phosphorylated by OsGSK2. BR treatment induces OsDLT dephosphorylation through inhibiting OsGSK2 kinase activity. *OsDLT* expression is negatively regulated by BR and its promoter is bound by OsBZR1 in vitro. Together, these finding suggest that in rice BR signaling, OsDLT and OsBZR1 carry out similar positive functions, while OsLIC plays a negative role. How BR balances the action of these three proteins to regulate rice growth remains unknown (Tong et al. 2009, 2012).

Extensive crosstalk between BRs and GAs in a wide range of biological processes has been reported (De Vleesschauwer et al. 2012). OsGSR1, a member of the GAST family in rice, is induced by GA and suppressed by BRs. OsGSR1 activates BR biosynthesis by directly interacting with DWARF1. Thus, OsGSR1 serves as a point of negative crosstalk between the GA and BR-signaling pathways (Wang et al. 2009). In addition, exogenous BR treatment enhances stability of OsSLR1, a GA signaling repressor in rice (De Vleesschauwer et al. 2012). Expression of *OsSLR1* increases in response to pathogen infection and BR treatment. However, the crosstalk between BRs and GA is likely complex, as demonstrated by the findings that treatment with BRs down-regulates four *DELLA* genes in cotton fiber cells (Hu 2011) and that in *Arabidopsis*, DELLAs directly interact with AtBZR1 and inhibit its DNA binding both in vitro and in vivo (Bai et al. 2012).

### G-proteins are involved in an alternative BR-signaling pathway in rice

The *d1* mutant that was originally identified as being defective in a positive regulator of GA signaling (Ueguchi-Tanaka et al. 2000), has now been shown to have altered BR signaling as well. The *d1* mutant displays the characteristic BR-related mutant phenotype including shortened second internodes, erect leaves, and constitutive photomorphogenetic growth in the dark. The *d1* mutant also exhibits decreased sensitivity to BR in many of the aspects examined such as lamina joint bending, coleoptile and second sheath elongation, and root growth inhibition. *D1* encodes rice heterotrimeric G-protein α subunit, RGA1, which has been suggested to play important roles in many signal transduction processes. BR treatment can repress the expression of BR biosynthetic genes in wild type, and this inhibition is impaired in the *OsBRI1* mutant *d61*. However, this feedback regulation functions normally in the *d1* mutant, and the amounts of BR intermediates in the *d1* mutant are not different with those in WT. Double mutant analysis showed no apparent epistasis between *d1* and *d61*-*7*. All of these results suggest that OsD1/RGA1 could be involved in an alternative BR-signaling pathway independent of OsBRI1 (Oki et al. 2009a, b; Wang et al. 2006).

Whether this putative signaling pathway is mediated through another plasma membrane-located BR receptor, such as OsBRL1 or OsBRL3, is still unclear so far. *OsBU1* (*BR upregulated 1*), encoding a HLH protein, is another positive regulator of BR responses. It is a primary response gene that participates in both the OsBRI1 and OsD1/RGA1 BR-signaling pathways (Oki et al. 2009a, b; Tanaka et al. 2009). Recently, an OsD1/RGA1 genetic interactor, *Taihu Dwarf 1* (*TUD1*), that encodes a functional U-box E3 ubiquitin ligase was reported (Fig. 1). Genetic, phenotypic, and physiological analyses have shown that *OsTUD1* is epistatic to *OsD1/RGA1* and the mutant is less sensitive to BR treatment. OsD1/RGA1 directly interacts with OsTUD1, which demonstrates that OsD1 and OsTUD1 act together to mediate a BR-signaling pathway. This supports the hypothesis that an OsD1/RGA1-mediated BR-signaling pathway acts in rice to influence plant growth and development (Hu et al. 2013). This idea is in agreement with the finding that *Arabidopsis* heterotrimeric G-protein α subunit (GPA1) is involved in the developmental regulation of BR signaling and biosynthesis (Gao et al. 2008). Although the basic signaling pathway appears to be highly conserved in plants, variation in signaling targets and the specifics in regulation may still underlie the various morphologies found in different species. In rice, there is some evidence for links between BRs and other hormones, such as GAs (Wang et al. 2009). BR-signaling network interactions with other hormone pathways are involved in yield component traits and stress responses (Hao et al. 2013).

### BR homeostasis in *Arabidopsis* and rice

**Fig. 3:**
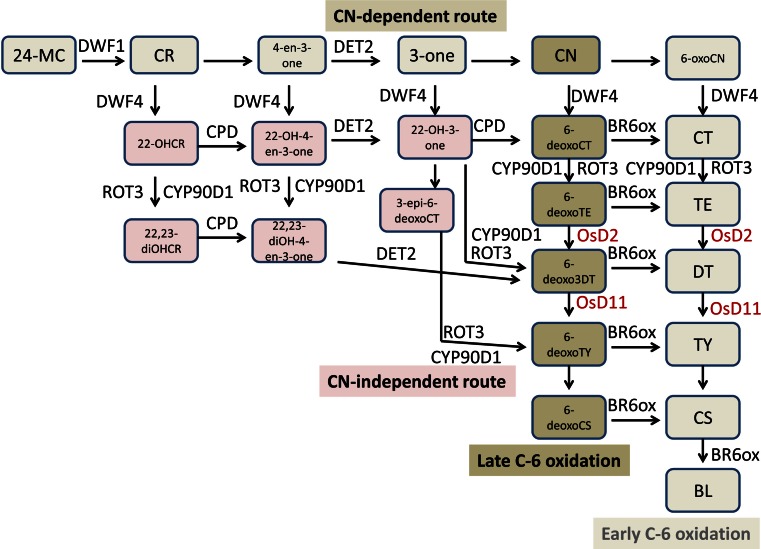
BR biosynthetic pathway and the enzymes involved in each reaction. Most of the enzymes shown are from *Arabidopsis*; those in *red* are the enzymes that have been identified only in rice (Choi et al. 1996; Fujioka et al. 2000; Hong et al. 2003, 2005; Kwon M 2005; Noguchi et al. 2000; Tanabe et al. 2005; Vriet et al. 2013). *CR* campesterol, *CN* campestanol, *CT* cathasterone, *TE* teasterone, *DT* dehydroteasterone, *TY* typhasterol, *CS* castasteronel, *BL* brassinolide

Over the past decades, extensive research has demonstrated the significance of BR homeostasis for normal plant growth. BRs are widely distributed throughout reproductive and vegetative plant tissues and do not travel over long distances in the plant (Symons et al. 2006). Thus, local biosynthesis of BRs is critical for regulation of downstream signaling. To date, almost all of the key enzymes involved in BR biosynthesis have been well characterized in both *Arabidopsis* and rice (Fig. 3). In *Arabidopsis*, the BR biosynthesis genes were identified mostly by forward genetics, including *DE*-*ETIOLATED 2* (*DET2*), *CONSTITUTIVE PHOTOMORPHOGENESIS AND DWARF* (*CPD*), *DWARF4*, *CYP724A1*, *ROT3* (*CYP90C1*), *CYP90D1*, *BR6OX1* (*CYP85A1*) and *BR6OX2* (*CYP85A2*) (Ohnishi et al. 2012); (Koka et al. 2000; Sakamoto 2006) (Choe et al. 1998); (Zhang et al. 2012a, b); (Ohnishi et al. 2006); (Bishop et al. 1999; Shimada et al. 2001). Most, if not all, BR biosynthetic enzymes may function as a multi-enzyme complex in the ER, enabling the biosynthesis of BL and related BRs by multiple routes (described as a ‘metabolic grid’ in Fujioka and Yokota 2003).

In rice, several BR biosynthetic enzymes have been identified by mutant screening. OsBRD2, a homolog of *Arabidopsis* DIMINUTO/DWARF1 (DIM/DWF1) catalyzes the conversion of 24-methylenecholesterol (24-MC) to CR (Hong et al. 2005). *OsD2* encodes a P450 (CYP90D2) similar to *Arabidopsis* CPD and DWF4 (Hong et al. 2003). CYP90D2 and CYP90D3 play redundant roles in multiple C-23 hydroxylation steps in the rice BR biosynthetic pathway (Hong et al. 2003; Sakamoto et al. 2012). *OsD11* encodes CYP724B1 and functions at the C-3 reduction step, catalyzing 6-deoxo 3DT and 3DT to 6-deoxoTY and TY (Tanabe et al. 2005). OsBRD1, a homolog of BR6OX, regulates multiple C-6 oxidation steps (Hong et al. 2002; Mori et al. 2002).

Plants have evolved strategic approaches to regulate BR homeostasis. BRs are transported within the cell from their site of biosynthesis in the ER to the PM (Symons et al. 2008). This transport might involve the formation of BR conjugates with fatty acids or glucose and/or the binding of BRs to transporter proteins (Sasse 2003). Other putative transport mechanisms might involve Sec14 cytosolic factor-mediated vesicle trafficking, and several Sec14 proteins have been identified as BR-regulated proteins in *Arabidopsis* (Deng et al. 2007), suggesting that there could be regulation of BR homeostasis at the level of transport. BR homeostasis is also regulated via a feedback loop in which the BR biosynthetic genes *DWF4*, *CPD*, *BR6OX1* and *ROT3* are down-regulated by the signaling components BZR1 and BES1 in *Arabidopsis*, and by OsBZR1 and OsDLT in rice (Wang et al. 2002; Yin et al. 2002). Other transcription factors reported to control BR biosynthesis and inactivation include TCP1, CESTA, BRX1, RAVL1 and Pra2 (Choe et al. 2001; Guo et al. 2010; Je et al. 2010; Kang et al. 2001; Mouchel et al. 2006; Poppenberger et al. 2011; Wang et al. 2001). A critical area remaining to be addressed is elucidating the molecular mechanisms that determine the bioactive levels of BRs in response to various developmental and environmental cues.

### BR and rice agronomic traits

#### Plant height

The high-yielding semi-dwarf varieties of wheat and rice combined with the application of nitrogen fertilizer contributed to the green revolution, which is a very important trait for high-yield breeding in crops. The reduction in plant height in semi-dwarf varieties led to improved harvest index (grain: straw ratio) and enhanced biomass production (Sakamoto and Matsuoka 2004). The rice stem consists of hollow internodes and jointed nodes, which are usually called culms, and to a large extent the stem contributes to the plant height. Based on the elongation pattern of internodes, rice dwarf mutants were previously categorized into five groups: dn, dm, d6, nl and sh (Hong et al. 2004).

BR-deficient and -insensitive mutants display a dwarf phenotype with a specific pattern of internode elongation. Usually, the second internode from the top is shortened completely in plants harboring severe BR mutant alleles and partially in those with mild BR mutant alleles, whereas elongation of the other internodes is affected very little (Yamamuro et al. 2000). This phenotype is known as dm-type dwarfism. OsBRI1 was characterized through the dwarf mutant *d61*, which has reduced sensitivity to BR. Mutants with a weak allele of *d61* fail to elongate the second internode (dm-type mutants) whereas those with the strong allele fail to elongate all internodes except the uppermost one (which is characteristic of d6-type dwarf mutants). OsBRI1 probably regulates internode elongation by inducing the formation of the intercalary meristem and longitudinal elongation of internode cells. It is presumed that internodes differ in their sensitivity to BRs. *OsBRI1* expression level is greater in the upmost and fourth internodes which allows to respond to the BR signal by inducing elongation. The dm-d6-type dwarfism caused by the severe allele of *d61* may be explained by the uppermost internode being exposed to more BRs than the fourth internode. Although BRs are not transported over long distances in the plant, it is possible that large quantities of BRs could move downward from the anthers to the lower organs, including the uppermost internode, and there induce internode elongation (Morinaka et al. 2006; Yamamuro et al. 2000). In addition to BR-insensitive mutants, dm-type dwarfism is also displayed by BR-biosynthetic mutants *d2* and *d11* (Hong et al. 2003; Tanabe et al. 2005). As mentioned above, the *d1* mutant (mutated in RGA1) was originally described as a GA signal transduction mutant. Interestingly, *d1* shows dm-type dwarfism, suggesting that a specific mechanism links BR and GA action in the second internode (Ashikari et al. 1999; Fujisawa et al. 1999; Urano et al. 2013).

#### Leaf angle

Leaf angle is an important agronomic trait associated with photosynthesis. It is well known that plants with erect leaves can capture more light for photosynthesis and enable more dense plantings with a higher leaf area index, all of which contribute to increase yields (Sinclair and Sheehy 1999). In rice, the contribution of lower leaves to photosynthesis is still significant even though the photosynthetic capacity of lower leaves is lower than that of upper leaves (Horton 2000). Erect leaves allow greater penetration of light to lower leaves and avoid the yield ceiling optimizing canopy photosynthesis.

Two main hormones, BRs and auxin, have been reported to be involved in determining leaf angle in rice (Nakamura et al. 2009). In physiological experiments, the degree of lamina inclination is a good indicator of the concentration of BRs in vivo (Wada et al. 1981). BR-deficient and -insensitive mutants show erect leaves, whereas overexpression of BR biosynthetic genes and signaling components increases leaf inclination (Bai et al. 2007; Hong et al. 2005; Yamamuro et al. 2000). For example, the *d61* mutant, which is defective in BR perception, displays erect leaves. In this case, longitudinal elongation of surface cells on the adaxial side of the lamina causes the lamina inclination. RAV-LIKE 1 (RAVL1) maintains BR homeostasis via the coordinated activation of *OsBRI1* and biosynthetic genes *D2, D11* and *BRD1* in rice, and the *ravl1* mutant has dark-green and erect leaves. The number and size of bulliform cells is also increased in the *ravl1* mutant, similar to in the *BR*-*deficient dwarf 1* (*brd1*) mutant (Hong et al. 2002; Je et al. 2010). OsBU1 controls bending of the lamina joint downstream of OsBRI1 and OsD1/RGA1 (Tanaka et al. 2009). A new member of the GRAS family, OsGRAS19, was reported to be a positive regulator in the BR-signaling pathway. *OsGRAS19* RNAi plants exhibited erect leaves and panicles and *OsGRAS19*-overexpressing plants displayed narrow leaves, larger leaf angles, and thin culms and panicle stems (Chen et al. 2013).

Other recent insights into the mechanism of leaf angle determination have emphasized the importance of auxin as well as BRs. *OsLC1* (*LEAF INCLINATION 1*) is transcribed in various tissues and encodes OsGH3-1, an indole-3-acetic acid (IAA) amido synthetase, whose homolog functions in maintaining the auxin homeostasis by conjugating excess IAA to amino acids in *Arabidopsis*. The dominant mutant *lc1*-*D* is insensitive to IAA and hypersensitive to exogenous BR, in agreement with the microarray data that suggests the altered transcription levels of genes involved in auxin signaling and BR biosynthesis. These results indicate that auxin homeostasis plays crucial roles in leaf inclination control (Zhao et al. 2012). The *OsLC2* (*LEAF INCLINATION 2*) gene encodes a putative vernalization insensitive 3-like protein. The *lc2* mutants display enlarged leaf angles with increased cell division in the adaxial side of lamina joint. *LC2* is response to various hormones such as abscisic acid, gibberellic acid, auxin, and BRs, and mainly expressed in the lamina joint (Zhao et al. 2010). *ili1*-*D* was identified as having a large leaf angle in a screen of rice T-DNA insertion mutants. *ili1*-*D* displays increased lamina joint bending and hypersensitivity to BR, which are caused by overexpression of the HLH transcription factor OsILI1. Another bHLH transcription factor, OsIBH1, an OsILI1-binding protein, inhibits cell elongation to decrease lamina joint bending. While OsLIC represses transcription of *OsILI1*, OsBZR1 represses *OsIBH1* as described above (Zhang et al. 2009a; Zhang et al. 2012a). These antagonistic transcription factors are good potential candidate genes for breeders hoping to design optimal rice architecture (Fig. 2) (Zhang et al. 2009a; Zhang et al. 2012a).

#### Tiller number

Tillering is an organegenesis process and one of the key agronomic traits. Tiller number per plant determines panicle number, a key component of grain yield. Rice tillering occurs in a two-stage process: the formation of an axillary bud at each leaf axil and its subsequent outgrowth (Li et al. 2003). Normally, a tiller bud arises from the axil of each leaf on the mother stem of a rice plant. Only those on the unelongated basal internodes have the potential to develop into tillers, whereas those formed on the elongated upper internodes become arrested when the mother stems begin to differentiate their own panicles (Wang and Li 2005). Generally, auxin and strigolactone as well as BRs are involved in tillering and branching in rice and *Arabidopsis* (Zuo and Li 2013). Rice tiller number and height are normally negatively related, i.e. dwarf plants usually have more tillers and vice versa, although there is no genetic evidence for a relationship between height and tillering. Most BR-related dwarf mutants do have more tillers than wild type with the exception of *dlt* (*dwarf and low*-*tillering*) (Tong et al. 2009). *dlt* is a semi-dwarf mutant with fewer tillers and dark-green, erect leaves. Lamina joint bending and coleoptile elongation assays showed that *dlt* has reduced sensitivity to BR. OsDLT is a GRAS family protein, and can repress the expression of some BR biosynthesis genes. BR treatment inhibits the transcription of *OsDLT*. OsBZR1, as a key transcription factor in BR signaling, can bind the promoter of *OsDLT* to repress its expression (Tong et al. 2009). It is possible that BR, acting through OsBZR1, regulates the expression of *OsDLT* and thereby controls rice tillering. In BR-deficient or -insensitive mutants, the activity of OsBZR1 would be inhibited by OsBIN2 phosphorylation, leading to high expression of *OsDLT*, thus inhibiting tillering. The increased expression of *OsDLT* in *d2* and *d11* supports this possibility (Tong et al. 2009). However, tissue-specific expression of the BR biosynthesis gene *DWF4* in rice can increase the tiller number, suggesting that BR promotes rice tillering (Wu et al. 2008). This result is contrast with the possibility that BR inhibits the expression of *OsDLT* to reduce tiller number. It may be explained by a complex interaction between BR and other hormones.

#### Stress response

It is known that BRs induce disease resistance in rice. Loss-of-function of OsGSK1 improves tolerance of cold, heat, salt and drought stresses compared with the wild type, but the underlying mechanisms remain elusive (Nakashita et al. 2003). Recently, several studies revealed that BR signaling and innate immunity-signaling pathways share multiple components including BAK1, BSK1 and BOTRYTIS-INDUCED KINASE 1 (BIK1) (Albrecht et al. 2012; Belkhadir et al. 2012; Lin et al. 2013; Shi et al. 2013) (Chinchilla et al. 2007; Heese et al. 2007; Wang 2012). Flagellin 22 (flg22) and chitin are well-characterized pathogen- and microbial-associated molecular patterns (PAMPs and MAMPs) that are important in biotic stress signaling. The binding of flg22 to its receptor FLS2 (flg-sensing 2) triggers an association and transphosphorylation with BAK1, thereby activating FLS2, similar to the BR-induction of BRI1 signaling. Then, the activated FLS2 phosphorylates BSK1 and BIK1 to trigger the target responses (Chinchilla et al. 2007; Lu et al. 2010; Sun et al. 2013; Zhang et al. 2010).

The coreceptor BAK1 functions both in BR-induced BRI1 signaling and flg22-induced FLS2 signaling. However, a recent study has suggested the existence of BAK1-independent immune signaling, and another study also provided evidence that BRs participate in MTI (MAMP-triggered immunity) through both BAK1-dependent and -independent pathways. The association of BRs with MTI responses depends on endogenous BR and BRI1 levels (Albrecht et al. 2012; Belkhadir et al. 2012). In addition, the effects of BRs on stress tolerance depend on the concentration of BRs applied to the plants. Excessive levels of BRs may have detrimental consequences because appropriate levels of BRs are required for optimal BR signaling. Through hormone crosstalk, both BRs and ABA promote stomatal closure, which is likely mediated by nitric oxide (NO). Indeed, NO was shown to mediate both ABA-induced stomatal closure and BR-induced ABA biosynthesis (Choudhary et al. 2012; Cui et al. 2011; Haubrick et al. 2006; Ribeiro et al. 2009; Zhang et al. 2011).

#### Grain size

BR-related mutants often showed altered seed size. *OsDWARF11* (*D11*) encodes a novel cytochrome (CYP724B1) and the *d11* mutant bears seeds of reduced length and equal width. The highest levels of *OsD11* expression were found in the internodes and the florets before flowering, and may correlate with the small-grain phenotype (Tanabe et al. 2005). Other mutants defective in BR biosynthesis, such as *d2* and *brd1*, bear small seeds that are shorter in both length and width (Hong et al. 2002, 2003). The *d1* mutant, which is deficient in RGA1 as described above, bears small seeds similar to those of *d11*, implying a potential relationship between BR signaling and G-protein-coupled pathways (Oki et al. 2009a, b). Rice plants overexpressing *OsILI1* and *OsBU1/ILI4* show enhanced bending of the lamina joint and increased grain size (Tanaka et al. 2009). The dominant short-grain mutant *Short grain 1* (*Sg1*) was identified via phenotypic screening of 13,000 rice activation-tagged lines. The causative gene, *OsSG1*, encodes a protein with unknown function that is preferentially expressed in roots and developing panicles. Overexpression of *OsSG1* in rice produces a phenotype with short grains and dwarfing reminiscent of BR-deficient mutants. Thus, OsSG1 appears to suppress responses to BRs. Despite shorter organs in the *SG1:OX* plants, cell size is not decreased. Therefore, SG1 decreases organ elongation by decreasing cell proliferation. In contrast to the *SG1:OX* plants, RNA interference knockdown plants in which *OsSG1* and a related gene, *OsSG1*-*LIKE PROTEIN1*, are down-regulated have longer grains and internodes in rachis branches than the wild type. Therefore, SG1 decreases responses to BRs and elongation of organs and the internodes of rachis branches through decreased cellular proliferation (Nakagawa et al. 2012).

#### Biomass and grain yield

Since their discovery in 1979, BRs have been considered promising compounds for application in agriculture and their economic value as yield-promoting agents was predicted by the early 1990s (Khripach et al. 2000). Treatment of rice plantlets with 24-epibrassinolide, a synthetic BR, leads to an increase of 22 % in seed fresh weight and of 31.5 % in seed dry weight per plant. BL treatment also increases plant growth rate, root size, and dry weight of root and stem (Zullo and Adam 2002). However, the high cost of synthetic BRs together with the variability of results has discouraged their use in agriculture. By contrast, modulating endogenous BR activity by direct manipulation of genes involved in either BR biosynthesis or signaling could allow for better crop yield and plant performance in a uniform and predictable manner (Divi and Krishna 2009).

BR-deficient and -insensitive mutants displaying dwarfism, erect leaves and reduced fertility are not a viable option for manipulating yield. However, the plants with slightly decreased BR levels or slightly suppressed BR signaling could be useful to increase yields significantly. For instance, the weak mutant allele *d61*-*7* causes a 35 % increase in biomass as compared to wild type at high planting density, although there is no difference in grain yield because of the small grain size in *d61*-*7* (Morinaka et al. 2006). To overcome the small-grain phenotype, Morinaka et al. used a co-suppression strategy to reduce the expression of endogenous *OsBRI1*, and two transgenic lines, BKD11 and BKD22, which display erect leaves and normal seed size, were selected. The estimated grain yield of these transformants is 30 % higher than that of wild type at high density. In addition, OsDWARF4 controls BR biosynthesis to influence lamina joint bending. The *osdwarf4*-*1* mutant shows erect leaves and normal reproductive development. Under high-density planting conditions, biomass and grain yield in *osdwarf4*-*1* are increased by nearly 40 and 26 %, respectively, over wild type (Sakamoto et al. 2006). These results demonstrate the feasibility of generating erect-leaf plants without defects in reproductive development by reducing the expression of the BR receptor or biosynthesis gene.

Photosynthesis and carbon fixation are also important in determining biomass. Rice plants severely deficient in or insensitive to BRs exhibit reduced leaf area and harvest indices, again making it seem unlikely that a reduction in overall BR levels could result in higher per-plant grain yields. However, manipulation of BR levels in specific parts of crop plants such as those affecting photosynthesis and assimilation could be one way to increase grain yields further. BRs regulate the initial carboxylation activity of ribulose-1, 5-bisphosphate carboxylase/oxygenase (Rubisco) and thereby influence photosynthetic CO_2_ assimilation (Yu et al. 2004). The expression of *DWARF4* (*DWF4)* is under tight transcriptional/post-transcriptional regulation to maintain BR homeostasis (Kim et al. 2006). In rice, ectopic overexpression of *Arabidopsis*, rice or maize *DWF4* under the control of the *S*-*ADENOSYLMETHIONINE SYNTHASE* promoter (p*AS*), which is active in the stems, leaves and roots of rice plants, results in between 15 and 44 % increases in grain yield. Microarray and photosynthetic analysis of transgenic plants revealed that enhanced CO_2_ assimilation, glucose accumulation are enhanced in the flag leaves, which causes increased assimilation of glucose into starch in the seed. These results further suggest that BRs stimulate the flow of assimilate (Wu et al. 2008). All of these findings demonstrate that BRs have the ability to control rice architecture and the flow of assimilate to influence yield. Therefore, genetic modulation of BR activity could be a practical strategy for generating high-yield transgenic rice.

#### Perspectives

Although recent research has elucidated most of the BR-signaling pathway in rice from the receptor OsBRI1 to the transcription factor OsBZR1, the key regulators of BR-induced responses and the mechanisms behind the pleiotropic actions of BRs remain poorly understood. So far, transcriptional analyses have identified hundreds of potential BR targets, shedding light on the complex activity of BRs in rice (Zhu et al. 2012). Targeted genetic engineering to modulate BR biosynthesis and BR signaling is a promising tool to improve biomass and stress tolerance in rice.

## Abbreviations

BR: Brassinosteroid
GA: Gibberellic acid
ABA: Abscisic acid
